# Temporal Trends in the Incidence and Mortality of Skin Malignant Melanoma in China from 1990 to 2019

**DOI:** 10.1155/2021/9989824

**Published:** 2021-08-24

**Authors:** Ruhai Bai, Hui Huang, Minmin Li, Meng Chu

**Affiliations:** ^1^School of Public Affairs, Nanjing University of Science and Technology, Nanjing 210094, Jiangsu, China; ^2^Department of Dermatology, The First Affiliated Hospital of Jinan University, Guangzhou 510632, Guangdong, China; ^3^Department of Infection Disease Control and Prevention, Shaanxi Provincial Center for Disease Control and Prevention, Xi'an, Shaanxi 710054, China; ^4^School of Public Health, Health Science Center, Xi'an Jiaotong University, Xi'an 710049, Shaanxi, China

## Abstract

**Purpose:**

Skin malignant melanoma (SMM) is one of the fastest-growing cancers in China, with a poor prognosis, high invasiveness, and high mortality rate. The aim of this study was to determine the long-term trends in the incidence and mortality of SMM in China between 1990 and 2019. *Patients and Methods*. Incidence and mortality data were extracted from the Global Burden of Disease Study 2019 and were analyzed using an age-period-cohort framework.

**Results:**

The annual incidence net drifts were 3.523% (95% confidence interval (CI): 3.318% to 3.728%) and 3.779% (95% CI: 3.585% to 3.974%) for males and females, respectively, while the corresponding annual net drifts of mortality were −0.754% (95% CI: −1.073% to −0.435%) and –0.826% (95% CI: −1.164% to −0.487%). The local drift from 1990 to 2019 was highest in males aged from 25 to 29 years. After controlling for period deviations in a single birth cohort, the SMM incidence and mortality increased exponentially with age for both sexes. Similar increasing monotonic trends were found for period and cohort effects on the incidence, while a declining trend was found for mortality.

**Conclusion:**

While the age-standardized mortality rate of SMM in China has decreased in both sexes over the past 30 years, the crude incidence rate, age-standardized incidence rate, and crude mortality rate have all increased. SMM may greatly threaten the health of the elderly in China due to the aging population. Appropriate changes should be made to raise the awareness, reduce the exposure to risk factors, and promote the early detection of SMM.

## 1. Introduction

Skin malignant melanoma (SMM) is the most aggressive and prevalent form of skin cancer that is characterized by a poor prognosis, frequent metastasis, and high mortality rate [1, 2]. Melanoma only accounts for 1-2% of all cancer cases worldwide and less than 5% of skin cancers, but is responsible for around 95% of deaths due to skin cancer [3]. Melanoma can also appear early in life and is the solid tumor that causes the greatest number of potential life-years lost [3, 4].

The global incidence of melanoma has been increasing rapidly recently [3, 5], from 2.01 per 100,000 person-years in 1990 to 3.75 per 100,000 person-years in 2019 [6]. Although the incidence and mortality of SMM in China are lower than the global average, they have recently increased rapidly to become one of the fastest-growing cancers in China [7]. SMM in China is more aggressive and has higher mortality rates compared with European and American populations [8]. The 5-year survival rate of metastatic patients is lower than 20% [9].

Previous investigations of SMM in China have often focused on the risk factors, clinical features, and prognosis [10, 11]. There have been some Chinese cancer-trend reports [12, 13] mentioning melanomas, but not specifically SMM. A few studies have compared the incidence and mortality between age groups, but these studies have not considered the impact of period and cohort effects. It is, therefore, necessary to conduct a comprehensive analysis of these limitations. This study aimed to use the 2019 Global Burden of Disease (GBD) data from 1990 to 2019 to determine the long-term trend in SMM incidence and mortality rates in China and use an age-period-cohort (APC) framework to analyze the corresponding effects on incidence and mortality rate between sexes. The present findings can provide guidance for etiology research of SMM morbidity and mortality, health resource allocation, and policy formulation to improve the prevention and treatment for high-risk groups.

## 2. Materials and Methods

### 2.1. Data Sources

The data analyzed in this study came from GBD 2019, which provides a systematic scientific assessment of published and publicly available data on incidence, prevalence, and mortality of disease and injury. GBD 2019 assesses the incidence, prevalence, mortality, years lived with disability, years of life lost, and disability-adjusted life-years for 369 diseases and injuries for different age groups and sexes, covering 204 countries and territories around the world from 1990 to 2019 [6]. GBD 2019 data on China mainly come from two sources: [14] surveillance data from the China Disease Surveillance Points system (covers 24.3% of the total population of the country since 2013) and Vital Registration data (accounting for roughly 8% of the national population) collected by the Chinese Center for Disease Control and Prevention [15]. These two systems are well designed and would provide a nationally representative picture of diseases in China [15, 16]. For GBD data, many steps are taken to enhance the data quality, with bias reduced to some extent compared with that of research using raw data [17]. This study was reported according to the Reporting of studies Conducted using Observational Routinely-collected health Data Statement (RECORD) [18]. SMM is often determined by the following diagnostic codes: ICD-9 code 172–172.9 and ICD-10 codes C43–C43.9, D03–D03.9, D22–D23.9, and D48.5. SMM incidence and mortality in this study were standardized using the global age-standardized population of GBD 2019.

Data sources for the incidence and mortality rate of SMM can be explored using an online tool produced by the Institute for Health Metrics and Evaluation (http://ghdx.healthdata.org/gbd-2019). The GBD 2019 data are freely available to the world's researchers and policymakers, and it uses deidentified, aggregated data; therefore, a waiver of informed consent was reviewed and approved by the University of Washington Institutional Review Board.

### 2.2. Statistical Analyses

An APC analysis aims to assess how age, period, and cohort effects contribute to outcomes. The age effect represents the differing outcome risks associated with different age groups; the period effect represents outcome variations over time that influence all age groups simultaneously, and the cohort effect is associated with outcome changes across groups with the same birth years [17]. The APC model was, therefore, used in this study to determine how age, period, and cohort effects influence the incidence and mortality of Chinese SMM patients.

The APC analysis was conducted by arranging incidence, mortality, and population data into consecutive 5-year periods from 1990 to 1994 (median 1992) to 2015–2019 (median 2017) and successive 5-year age intervals from 0–4 years to 90–94 years [19]. SMM incidence and mortality in patients aged >95 years were not considered in this study since those in this age group are divided into a single group in the GBD database. Parameters were estimated using the APC web tool (Biostatistics branch, National Cancer Institute, Bethesda, MD) [20]. Four functions were estimated using APC analysis. The first was net drift, corresponding to the overall log-linear trend in calendar periods and birth cohorts that indicate the overall annual percentage change. The second was local drift, corresponding to the age-group-specific log-linear trend in calendar periods and birth cohorts, which indicates annual percentage changes for each age group. The third was longitudinal age curves, which indicate the fitted longitudinal age-specific rates in the reference cohort after adjusting for period deviations. The fourth was cohort (or period) relative risks (RRs), which represent the cohort (or period) RR adjusted for age and nonlinear period (or cohort) effects in a cohort (or period), against the reference cohort [17]. In the APC analysis, the median age group, period, and birth cohort were defined as the reference.

Wald chi-square tests were used for the significance tests of the estimating functions. All statistical tests were two sided and were considered statistically significant at *P* *<* 0.05.

## 3. Results

### 3.1. Trends in SMM Incidence and Mortality Rates by Sex from 1990 to 2019

**Figure 1:**
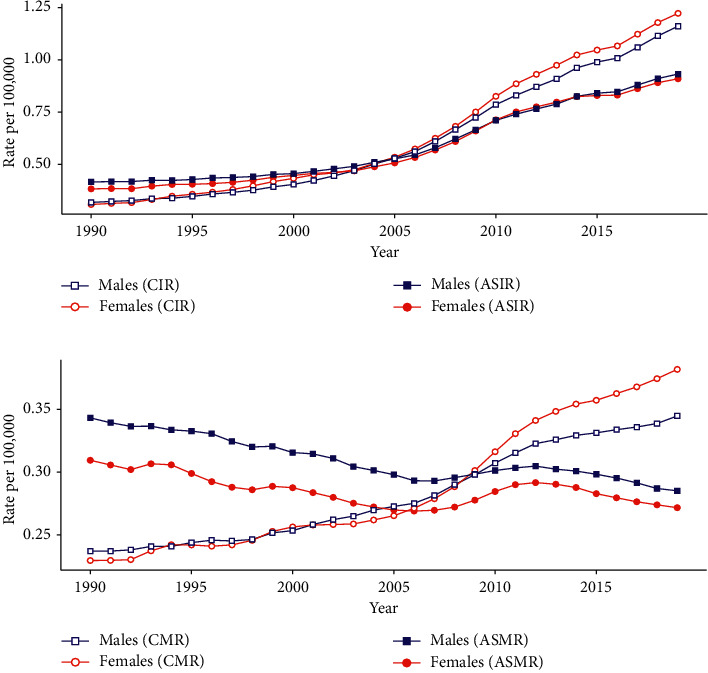
Trends in age-standardized and crude incidence and mortality rates per 100,000 person-years according to sex for skin malignant melanoma (SMM) in China from 1990 to 2019: (a) ASIR and CIR and (b) ASMR and CMR. The GBD 2019 global age-standard population data were analyzed. SMM: skin malignant melanoma; ASIR: age-standardized incidence rate; ASMR: age-standardized mortality rate; CIR: crude incidence rate; CMR: crude mortality rate; GBD: global burden of disease study.

Figures 1(a) and 1(b) display the trends in crude incidence rate (CIR), age-standardized incidence rate (ASIR), crude mortality rate (CMR), and age-standardized mortality rate (ASMR) for SMM by sex for the period from 1990 to 2019.  Figure 1(a) suggests that the CIR for SMM in China showed increasing trends for both sexes, from 0.318 to 1.161 and from 0.308 to 1.222 per 100,000 person-years for males and females, respectively. The ASIRs also showed increasing trends, from 0.416 to 0.932 and from 0.383 to 0.909 per 100,000 person-years for males and females, respectively. The ASIR for SMM increased by 124.04% and 137.34% in males and females, respectively.  Figure 1(b) displays the changes in the SMM mortality rates of Chinese males and females and suggests that although the CMRs in both sexes showed increasing trends, from 0.237 to 0.345 and from 0.230 to 0.382 per 100,000 person-years for males and females, respectively, the ASMRs in both sexes had general decreasing trends, from 0.343 to 0.285 and from 0.309 to 0.271 per 100,000 person-years for males and females, respectively. It should be noted that the ASMRs for SMM increased slightly from 2006 to 2012 for both sexes. The ASMR for SMM reduced from 1990 to 2019 by16.91% and 12.30% in males and females, respectively.

### 3.2. Local Drift with Net Drift Values for SMM Incidence and Mortality Rates in China

**Figure 2:**
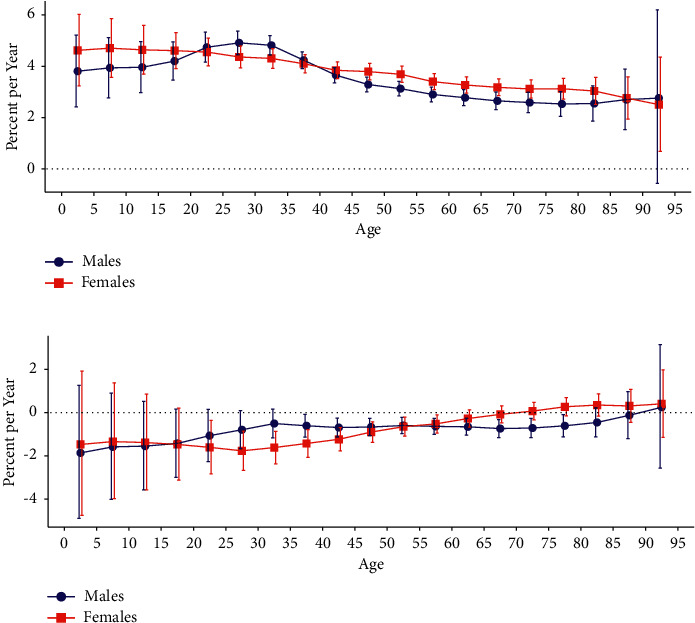
Local drift with net drift values for SMM incidence and mortality in China: (a) incidence and (b) mortality. Age-group-specific annual percentage change (local drift) with the overall annual percentage change (net drift) in SMM incidence and mortality rates and their corresponding 95% CI are shown. SMM: skin malignant melanoma; CI: confidence interval.

Figure 2 displays the net drift (overall annual percentage change) and local drifts (annual percentage changes for each age group) for SMM incidence and mortality rates in China. The annual net drifts for SMM incidence were 3.523% (95% confidence interval (CI): 3.318% to 3.728%) and 3.779% (95% CI: 3.585% to 3.974%) for males and females, respectively.  Figure 2(a) shows that the local drift values for SMM incidence were above 0 in all age groups for females (*P* *<* *0.05*) and the highest in those aged 5–9 years at 4.705% (95% CI, 3.569% to 5.853%). The local drift values for male SMM incidence were above 0 in all age groups (*P* *<* *0.05*) except those aged 90–94 years and the highest in those aged 25–29 years at 4.912% (95% CI: 4.455% to 5.370%). Overall, the SMM incidence increased more for females than for males in all age groups except for those aged 20–24 and 35–39 years. The annual percentage change was an increase of more than 4% in those aged 0–4 and 35–39 years for both sexes. The annual net drifts of SMM mortality were −0.754% (95% CI: −1.073% to −0.435%) and −0.826% (95% CI: −1.164% to −0.487%) for males and females, respectively, and the local drift values were below 0 in males aged 35–39 and 75–79 years and in females aged 20–24 and 55–59 years (*P* < 0.05).

### 3.3. Longitudinal Age Curves of SMM Incidence and Mortality by Sex in China

**Figure 3:**
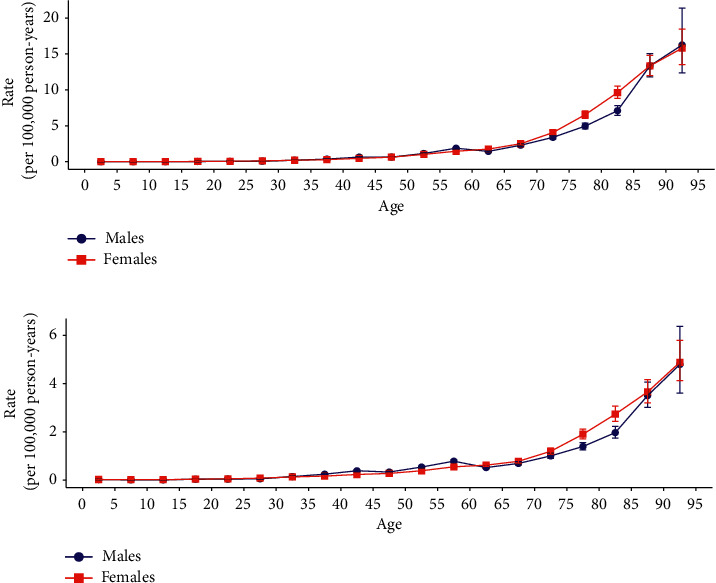
Longitudinal age curves of SMM incidence and mortality in China: (a) incidence and (b) mortality. Fitted longitudinal age-specific SMM incidence and mortality rates (per 100,000 person-years) and the corresponding 95% CI (some of which are too narrow to see in the figure) are shown. SMM: skin malignant melanoma; CI: confidence interval.

The longitudinal age curves of SMM incidence and mortality by sex are displayed in Figure 3. Males and females in the same birth cohort had a higher risk of SMM morbidity and mortality at ages of 0–4 and 90–94 years. Further estimations based on the longitudinal age curves indicated that both sexes exhibited exponential distributions. The incidence curves could be expressed as rate = 0.007 × *e*0.088 × mean_age (*R*^2^ = 0.994) and rate = 0.008 × *e*0.086 × mean_age (*R*^2^ = 0.985) for males and females, respectively, while the corresponding mortality rates were 0.014 × *e*0.062 × mean_age (*R*^2^ = 0.955) and 0.015 × *e*0.062 × mean_age (*R*^2^ = 0.988).

### 3.4. Period and Cohort RRs of SMM Incidence and Mortality Rate by Sex in China

**Figure 4:**
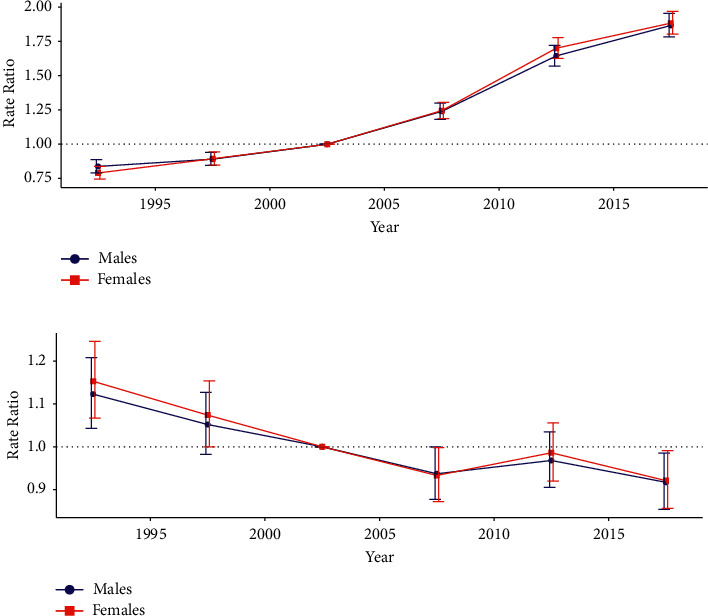
Period RRs of SMM incidence and mortality rates by sex in China: (a) incidence and (b) mortality. The RR of each period compared with the reference year (year 2000–2004) adjusted for age and nonlinear cohort effects and the corresponding 95% CI are shown. RRs: relative risks; SMM: skin malignant melanoma; CI: confidence interval.

**Figure 5:**
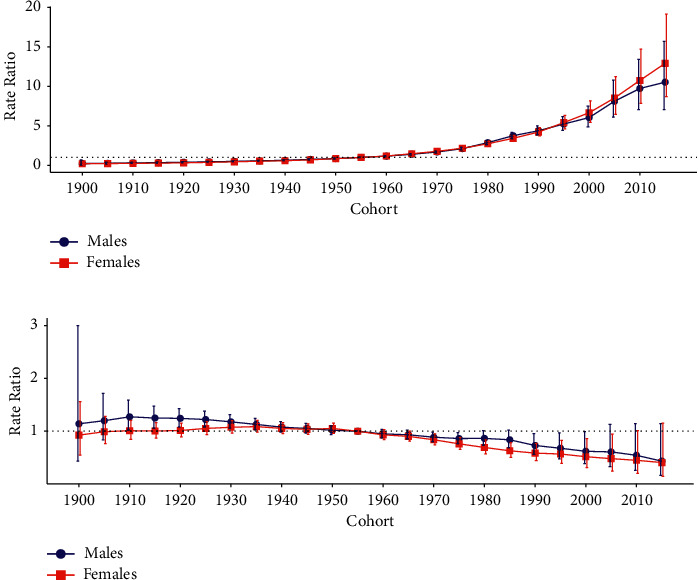
Cohort RRs of SMM incidence and mortality rate by sex in China: (a) incidence and (b) mortality. The RR of each cohort compared with the reference cohort (cohort 1955) adjusted for age and nonlinear period effects and the corresponding 95% CI are shown. RRs: relative risks; SMM: skin malignant melanoma; CI: confidence interval.

Figure 4 displays the estimated period and cohort RRs for SMM incidence and mortality rate by sex. The period RRs of SMM incidence and mortality had similar patterns for both sexes (Figures 4(a) and 4(b)), with the incidence increasing significantly after the reference period (year 2000–2004) and the mortality decreasing after the reference period (year 2000–2004). The cohort RRs for incidence rate showed an overall increasing pattern from earlier to later birth cohorts for both sexes (Figure 5(a)), while the cohort RRs for the mortality rate had a decreasing pattern after the reference birth cohort (year 1955) for both sexes (Figure 5(b)).

## 4. Discussion

To our knowledge, this is the first study to investigate the long-term trends in the incidence and mortality of SMM in China and examine age-, period-, and cohort-specified effects using an APC framework. The present results indicate that the CIR and ASIR for SMM in Chinese males and females generally increased between 1990 and 2019, with increases in the SMM incidence rate for every age group except among those aged 90–94 years. Although the SMM CMR of Chinese males and females generally increased between 1990 and 2019, their SMM ASMR values showed overall decreasing trends among males aged 35–39 and 75–79 years and females aged 20–24 and 55–59 years.

This study found a difference between the SMM CMR and ASMR, with the aging society considered to play an important role in this difference. China was considered an aging society from 2000, and the size of the elderly population is currently growing much more rapidly than any other age group [21]. The number of seniors aged 60–79 and 80–94 years in China increased from 1953 to 2010 by about 3.5–6.6 times and 9.3–28.23 times, respectively. In 2010, people older than 60 and 65 years accounted for 13.3% and 8.9% of the total Chinese population, respectively [22], indicating rapid aging [21]. Age is an important demographic risk factor affecting the occurrence of and death due to SMM [23]. Based on the latest GBD 2019 data, our results indicate that SMM occurrence and mortality in the same birth cohort increase exponentially with age after adjusting for period deviation. Figure 3 displays the concentrated risk of SMM in China in the elderly stage, which may be related to the impaired immune response of the elderly to malignant tumors and infections [24]. Although the SMM ASMR has declined due to China's economic development [25], progression in medical technology [2, 26], and per-capita education level [26], the SMM CMR of the elderly has continued to increase due to increasing size of their population. Zeng and Wang [27] and Chen and Liu [28] estimated that by 2050, there will be 329 million people older than 65 years, accounting for 25% of the Chinese population, and the growth rate of people older than 80 years will be even greater, increasing from 0.9% in 2000 to 7.2% in 2050. Moreover, there will be twice as many people older than 65 years in rural than in urban areas. Considering the current trends, we predict that the SMM CMR of Chinese males and females is likely to continue to increase in the future [24].

It is noteworthy that the risk of melanoma is significantly higher in males than in females among Chinese people aged 20–40 years, whereas it is significantly higher in females than in males among the United States population younger than 45 years [23, 29]. Indoor tanning is a likely factor for the diverging trends in males and females younger than 50 years in the United States [29], while in China, indoor tanning is less popular. The risk might be higher in young Chinese males than females due to Chinese adult males tending to participate in more outdoor activities than females [30] and, hence, being exposed to more ultraviolet radiation [31]. Moreover, males are reportedly less aware of the hazards of excessive sun exposure and the correct protection measures (e.g., using a parasol) than females and are more likely to ignore public health education [31]. When outdoors, males are less likely to take appropriate sun protection measures [31], leading to the skin absorbing more ultraviolet radiation. Males are also more sensitive to ultraviolet radiation [32], which is responsible for 37% of melanomas in males but less than 9% of those in females [32]. All of these abovementioned factors may contribute to the morbidity risk for Chinese males aged 20–40 years being higher than that for females.

While period and cohort effects can be estimated separately as the period RR and cohort RR using certain restrictions, it is difficult to interpret them separately in the real world. This is because the period effect often influences age groups differently when it is applied to all age groups simultaneously, leading to the cohort effect [17]. It is, therefore, necessary to comprehensively discuss the possible reasons for the period and cohort effect trends. In this study, the period and cohort effects related to SMM incidence showed increasing trends, while the period and cohort effects for SMM mortality generally showed decreasing trends.

The increased SMM incidence may be related to the increase in the detection rate of melanomas in China. Compared with other cancers, malignant melanoma is characterized by skin localization, which allows for its early detection through noninvasive methods [33]. The detection and diagnosis rates of melanoma in China have recently been increasing with the developments in the economy and medical technology [34]. Simultaneously, the public's healthcare awareness has continuously progressed with improvements in education levels, as has the understanding of the early warning signs of diseases [26]. The number of people undergoing physical examinations has increased each year due to the rapid development of physical examination institutions and community service centers, which have also greatly increased the detection rate for the disease, therefore increasing the period and cohort RRs of the disease risk [35]. Although the increased diagnosis rate of early melanoma also leads to a rapid increase in the incidence, the Breslow thickness of the early lesions is usually shallow and the prognosis is often better [35]. This may also explain why the period and cohort RRs of incidence were found to be increasing while the mortality RRs were decreasing.

From a macroperspective, the increase in SMM risk is somewhat related to the increase in natural ultraviolet radiation. The ozone layer located in the stratosphere (10–50 km above sea level) absorbs all UVC and most UVB and UVA [36]. The destruction of the ozone layer increases human skin exposure to ultraviolet radiation [37]. Studies have indicated that a 1% reduction in the ozone layer is equivalent to a 1-2% increase in the melanoma mortality rate [37], and it has been predicted that there will be a significant thinning of the ozone layer by 2065, which may lead to a very high incidence of skin cancer by 2100 [38]. Strong intermittent sun exposure can also significantly increase the melanoma risk [39]. Although previous studies have indicated that increases in melanoma incidence may also be related to increased use of indoor tanning equipment [29], the use of such equipment is less common in China, which, therefore, reduces its impact on the health of the population.

Primary prevention and early detection are crucial to addressing the health threat and economic burden of SMM [40]. We recommend that the general population should take measures to minimize their exposure to risk factors, such as avoiding exposure to intermittent high-dose sunlight as well as other types of ultraviolet radiation. Studies have indicated that although correctly using sunscreen can reduce the SMM incidence [40], traditional protection methods (e.g., wearing protective clothing and avoiding sun exposure or prolonged sunbathing) are more effective in reducing the melanoma risk [40, 41], and these measures cannot be replaced by sunscreen use [42]. To optimize protection from the damaging effects of the sun, we should, therefore, not just “slop on the sunscreen” but also “slip on a t-shirt, slap on a hat, seek shade, and slide on sunglasses.” Importantly, these measures should be implemented together, not just one or some of them [42]. Regular screening is recommended for elderly people and high-risk groups who are susceptible to melanoma (e.g., people with fair skin, multiple atypical moles, or a family history of melanoma) with the purpose of diagnosing thin melanomas early and reducing the associated mortality [43]. Germany recommends regular skin cancer screening for people older than 35 years, while in the United States, it is not recommended [44]. However, some skin cancer prevention programs in the United States have had beneficial impacts on early diagnosis and treatment, and the survival rate at 5 years after a melanoma diagnosis increased from 50% in 1950 to 90% in 1990 [3].

There are notable limitations to our study. First, the Chinese SMM data used in this study are only macroestimates for the country. Like other APC analyses, there was an inevitability of ecological fallacy because interpreted population results are not necessarily valid for individuals. Related hypotheses from this study, therefore, still need further confirmation in future individual-based studies. Second, the limited data meant that we did not distinguish between urban and rural areas for the SMM incidence and mortality in China. Considering that China has a typical urban-rural dual structure, it is necessary to analyze the differences between urban and rural SMM cases in the future.

## 5. Conclusions

In summary, although the ASMR of SMM in Chinese males and females has shown a downward trend over the past 30 years, the CIR, ASIR, and CMR have all exhibited upward trends. Our APC analysis indicated that the SMM incidence and mortality rates in the same birth cohort increased exponentially with age for both sexes after controlling for period deviations. Considering that early SMM detection can effectively improve the overall survival and cure rates of patients [39], it is necessary to strengthen the knowledge that the Chinese public has of the harms caused by melanoma. The general population should reduce their exposure to ultraviolet radiation using techniques such as avoiding exposure to intermittent high-dose sunlight as well as other types of ultraviolet radiation and wearing protective clothing [39]. High-risk populations (e.g., severe sunburn, skin cancer, pigmented nevus, and chronic inflammation of the acral skin) [43] should be regularly screened and visit hospitals if necessary, with the aim of diagnosing melanoma with a low invasion depth early and, thereby, reducing mortality [43].

## Data Availability

The datasets generated and/or analyzed during the current study are available in the Institute for Health Metrics and Evaluation: http://ghdx.healthdata.org/gbd-2019.

## Abbreviations

APC: Age-period-cohort
ASIR: Age-standardized incidence rate
ASMR: Age-standardized mortality rate
CI: Confidence interval
CIR: Crude incidence rate
CMR: Crude mortality rate
GBD: Global burden of disease
RECORD: Reporting of studies Conducted using Observational Routinely-collected health Data Statement
RRs: Relative risks
SMM: Skin malignant melanoma
UV: Ultraviolet.
